# Trajectory modelling of ambulatory care sensitive conditions in Finland in 1996–2013: assessing the development of equity in primary health care through clustering of geographic areas – an observational retrospective study

**DOI:** 10.1186/s12913-019-4449-7

**Published:** 2019-09-04

**Authors:** Markku Satokangas, Sonja Lumme, Martti Arffman, Ilmo Keskimäki

**Affiliations:** 1 Social and Health Systems Research Unit, National Institute for Health and Welfare, P.O. Box 30, 00271 Helsinki, Finland; 2 Department of General Practice and Primary Health Care, Network of Academic Health Centres, University of Helsinki, Helsinki, Finland; 3 Health Stations, Department of Social Services and Health Care, City of Helsinki, Finland; 4 Faculty of Social Sciences, Tampere University, Tampere, Finland

**Keywords:** Development of health equity, Ambulatory care sensitive conditions, Primary care, Inpatient hospitalisation, Group-based trajectory modelling

## Abstract

**Background:**

Due to stagnating resources and an increase in staff workload, the quality of Finnish primary health care (PHC) is claimed to have deteriorated slowly. With a decentralised PHC organisation and lack of national stewardship, it is likely that municipalities have adopted different coping strategies, predisposing them to geographic disparities. To assess whether these disparities emerge, we analysed health centre area trajectories in hospitalisations due to ambulatory care sensitive conditions (ACSCs).

**Methods:**

ACSCs, a proxy for PHC quality, comprises conditions in which hospitalisation could be avoided by timely care. We obtained ACSCs of the total Finnish population aged ≥20 for the years 1996–2013 from the Finnish Hospital Discharge Register, and divided them into subgroups of acute, chronic and vaccine-preventable causes, and calculated annual age-standardised ACSC rates by gender in health centre areas. Using these rates, we conducted trajectory analyses for identifying health centre area clusters using group-based trajectory modelling. Further, we applied area-level factors to describe the distribution of health centre areas on these trajectories.

**Results:**

Three trajectories – and thus separate clusters of health centre areas – emerged with different levels and trends of ACSC rates. During the study period, chronic ACSC rates decreased (40–63%) within each of the clusters, acute ACSC rates remained stable and vaccine-preventable ACSC rates increased (1–41%). While disparities in rate differences in chronic ACSC rates between trajectories narrowed, in the two other ACSC subgroups they increased. Disparities in standardised rate ratios increased in vaccine-preventable and acute ACSC rates between northern cluster and the two other clusters. Compared to the south-western cluster, 13–16% of health centre areas, in rural northern cluster, had 47–92% higher ACSC rates – but also the highest level of morbidity, most limitations on activities of daily living and highest PHC inpatient ward usage as well as the lowest education levels and private health and dental care usage.

**Conclusions:**

We identified three differing trajectories of time trends for ACSC rates, suggesting that the quality of care, particularly in northern Finland health centre areas, may have lagged behind the general improvements. This calls for further investments to strengthen rural area PHC.

**Electronic supplementary material:**

The online version of this article (10.1186/s12913-019-4449-7) contains supplementary material, which is available to authorized users.

## Background

In recent decades, Finnish public primary health care (PHC) has been suspected of slowly deteriorating – especially if compared to other sectors of the Finnish health care system, i.e. specialist care and occupational and private health care. In the absence of systematic PHC performance and quality indicators, this argument has been based on statistics regarding long waiting times [1], stagnating financial resources [2], stagnating numbers of both physicians and other personnel [3], a decrease in the number of GP consultations [4] and an increase in GP job strain and workload [1, 5]. This situation has persisted, although improving access to and quality of PHC has been referred to among the main policy measures in reducing Finnish health inequities [6]. As PHC has been found to reduce the ill effects of income inequality on health [7], the suspected deterioration could eventually have a negative impact on the already existing health inequities in Finland [8]. We found internationally no paper that would present a comprehensive approach to assess – and either to support or contradict – this kind of argument of PHC deterioration over time.

Whether or not this deterioration exists, in comparison to most European countries Finland has a strong, nationwide public PHC funded through taxes and operating on the principle of universal access [9]. Unlike in the other Nordic countries, Finland has lower number of physicians than the EU average – over third of them GPs [10]. The number of nurses is high as in the other Nordic countries. Though Finns have the EU average of life-expectancy and good perceived health, they also report one of the EUs highest proportions with chronic conditions and disabilities [10]. Finnish PHC acts as a gatekeeper to specialist care, which takes place mainly in public hospitals. Further, a hospital serves mainly geographically nearby municipalities in its hospital district. The national level of avoidable hospital admissions for five chronic conditions is slightly lower than the EU average [10]. The nationwide network of municipal health centres, the foundation of the Finnish public PHC was built in the 1970s, funded with state subsidies earmarked to PHC [11]. However, amidst the difficult recession in the early 1990s the state’s economic control over PHC services was abolished: the level of state subsidies was reduced and their earmarking was removed [1]. Simultaneously, the funding of specialist care was to be decided between municipalities, joint municipal authorities and hospital districts [11]. While funding both PHC and specialist care, individual municipalities have had increased autonomy in terms of organising and financing the first, but only minor control over the organising and the expenditures of the latter. Since these changes, specialist care has been able to secure its funding and its attractiveness as an employer, while municipal PHC has had to endure cost savings. After recovering from the recession, Finland has had a continuous physician shortage, with PHC having the least favourable position to compete for the workforce. In 2013 PHC in Finland, with approximately 5.4 million inhabitants, was organised independently in 89 municipalities and through 62 joint municipal authorities in the rest of the 215 municipalities. Due to this decentralisation and a lack of national stewardship with respect to PHC, it is likely that the stagnation of resources and strategies for coping with it have differed between municipalities. Thus, if the suspected deterioration exists, it might present itself in the form of increasing geographic health inequities – thus challenging the achievement of the national goal of health equity [12]. This necessitates the assessment of Finnish PHC performance by analysing over time geographic distribution in health quality outcomes.

This study aimed to identify any clusters of geographic areas where performance of PHC either develops clearly better or worse than elsewhere in Finland. To achieve this, we assessed over time the geographical distribution of Ambulatory Care Sensitive Condition (ACSC) rates in Finland for the years 1996–2013 using group-based trajectory modelling (GBTM). This approach to analyse geographic disparities distributes health centre areas (individual municipalities or joint municipal authorities) to trajectories, depending on the level and development of ACSC rates in these areas. The use of GBTM allowed us to identify homogenous clusters of developmental trajectories of ACSC rates by areas [13]. Further, we describe this distribution with area-level factors. These include health-care-dependant factors, the socioeconomic characteristics of the population, municipal characteristics and other health-related factors. Our hypothesis was that the suspected slow deterioration of PHC, due to decentralisation and a lack of national stewardship, would present itself as a geographical polarization of the health of the population between municipalities. Further, we hypothesised that the increasing proportion of elderly people, number of GP consultations and persons in a low socioeconomic position (SEP), as well as elevated morbidity or limitations in activities of daily living (ADL), would associate with an increased level of ACSC rates. There is a clear geographic inequity in morbidity among Finns, which we assumed to affect the ACSC rates: population of southern Finland is healthier than population of the eastern and northern Finland [14]. To the best of our knowledge no previous study has applied GBTM to analyse the geographical clustering of over time development of ACSC rates.

## Methods

### Data collection

To explore the extent of geographic variation and its temporal development in PHC, this retrospective observational study utilised routinely collected hospitalisation data to assess trajectories for ACSCs. This proxy indicator of PHC quality and performance is comprised of the conditions under which hospitalisations could possibly be prevented via the timely function of PHC. No single, universal list of ACSC conditions exists internationally as hospitalisation criteria vary between countries and health care systems [15]. As Finland has no validated list of ACSC conditions (for example through a Delphi process [16]), we applied its UK definition to maintain some international comparability [17]. However, eventual abandoning of ICD-9 codes from Finnish hospitalisation data in 2011 (previously alongside the ICD-10 codes) resulted in systematic drop of ACSC levels. This occurred due to unspecified pneumonia (J18.9), which was included before 2011 as it converts into the same Finnish ICD-9 code (485) as the diagnose J18.8 included in the UK definition. We chose to include J18.9 to maintain backward compatibility. In preliminary analyses, this single diagnosis covered three-fourths of all pneumonia hospitalisations [data not shown].

We obtained data on the hospitalisations due to ACSCs for the total Finnish population aged ≥20 for the years 1996–2013 from the Finnish Hospital Discharge Register, maintained by the National Institute for Health and Welfare (THL). To account for hospital transfers, we combined two consecutive ACSC hospitalisations (separated by only 1 day) to a single hospitalisation with the diagnose of the first one. To cover different preventive strategies, as proposed by previous studies [18, 19], we subcategorised the ACSC hospitalisations as either acute, chronic or vaccine-preventable (Additional file 1) [20]. Further, we analysed these subgroups by gender and geographic areas due to different levels and disease patterns of ACSCs in men and women [21]. The applied area unit was health centre areas: a total of 131 geographical areas, which represented either a single municipality or a consolidation of 2–8 small municipalities. In the latter case, PHC was organised by a joint authority running the local health centre organisation. Populations in these areas varied between 5200 and 612,700, with a median population of 22,600 in 2013.

We chose to include area-level descriptive factors, which have been suggested in previous studies assessing ACSCs, such as SEP [18], morbidity [22] and limitations in ADL [23]. Also, we included factors related to the usage of and access to PHC as well as factors describing the structure of municipalities – as these might be associated with some of the geographic differences in ACSC distribution. Table 1 summarises the included descriptive factors, which were collected annually for 1996–2013 from Sotkanet.fi, a Statistics and Indicator Bank maintained by THL [24] – expect for the pensioner’s care allowance which was collected from the Kelasto.fi, a statistical database maintained by the Social Insurance Institution of Finland [25]. The Research Ethics Committee of the National Institute for Health and Welfare provided ethical consent for the study.

**Table 1:** Area-level descriptive factors by their level of analysis

*List of variables*	*Comments*
*Health care -dependant factors*	
GP consultations per 1000 inhabitants	
Primary and secondary care expenditures	
Inpatient PHC periods per 1000 inhabitants	
Specialised somatic inpatient care periods per 1000 inhabitants	
Dentist visits in health centres per 1000 inhabitants	
Reimbursed private dental care visits per 1000 inhabitants	
Proportion of inhabitants reimbursed for private health care use	
*Sociodemographic characteristics of the populations*	
Proportions of inhabitants aged ≥65	
Ratio of people aged < 15 and > 64 to every hundred people aged 15–64	
Proportion of inhabitants with a tertiary education	
Average years of education after primary education	
Proportion of low-income households	
Proportion of inhabitants receiving basic social assistance	
Ratio of unemployed to total workforce	
*Municipal characteristics*	
Type of municipality	Either an individual municipality or a consolidation of municipalities
Proportion of inhabitants in the largest municipality	
Degree of urbanisation	
*Other health-related factors*	
Potential years of life lost	
Pensioner’s care allowance of recipients aged ≥65 per 1000 inhabitants of the same age	Used as a proxy for both morbidity and limitations in ADL, as these are the main requirements for the allowance.
THL’s morbidity index	Comprises cancer, coronary heart disease, cerebrovascular diseases, musculoskeletal diseases, mental health problems, accidents and dementia [14].

### Statistical analysis

We calculated annual age-standardised rates/100,000 person years in health centre areas by applying the direct method of standardisation and the European Standard Population [26]. No missing data or small cells existed within the age-standardised rates. To assess the development and clustering of these rates in the health centre areas, we applied group-based trajectory modelling [27]. In other words, with this modelling we identified health centre areas and grouped them into separate clusters according to the similarity of both their level and development of ACSC rates. The purpose of the analysis was to identify different developmental paths of ACSC rates between health centre areas which might go unnoticed with other statistical methods. With a multi-trajectory model, we analysed the distribution of the rates of three ACSC subgroups (acute, chronic and vaccine-preventable conditions) simultaneously within a single model [28]. The goodness-of-fit of the trajectory models was tested using Bayesian information criterion (BIC) [29]. Based on this approach, we chose models against the alternative ones where the evidence was “very strong” (BIC difference > 10) favouring the chosen trajectory models. Further, we calculated standardised rate ratios (SRRs) and rate differences (RDs) for these clusters of health centre areas. We tested the changes in annual trends of SRRs and RDs with linear regression model. Further, we analysed whether over time the development of area-level factors over time would associate with the distribution of health centre areas in the trajectories using an autoregressive Generalized Estimating Equation (GEE) model [30]. In other words, we tested whether any of these area-level factors had similar developments within a group of health centre areas over time along a single trajectory and that the finding was consistent for each of the trajectories. The differences of group averages in area-level descriptive factors between clusters were tested with ANOVA. The statistical analyses were performed using the TRAJ [31] and GEE procedures of the SAS system for Windows, release version 9.3 (SAS Institute, Cary, NC). The results were then combined with spatial information from Statistics Finland [32] using ArcGIS for Windows, release version 10.3.1 (Environmental Systems Research Institute, Redlands, CA).

## Results

Altogether, we identified 123,975 hospitalisations due to ACSCs in 1996, and 99,684 in 2013. Overall, the total ACSC rates decreased throughout the country, mainly due to a reduction in chronic ACSC rates. The vaccine-preventable ACSC rates slightly increased. In 1996 the vaccine-preventable ACSC rate in men for the whole country was 1070 per 100,000 person years, and 1100 in 2013. The corresponding chronic ACSC rates were 3470 in 1996 and 1280 in 2013; and acute ACSC rates 820 in 1996 and 680 in 2013. In women the corresponding values were: for vaccine-preventable ACSC rates 490 in 1996 and 610 in 2013; for chronic ACSC rates 2240 in 1996 and 950 in 2013; and for acute ACSC rates 670 in 1996 and 650 in 2013. The annual means and ranges of ACSC rates in health centre areas are presented in Additional file 2. Women had lower ACSC rates than men for chronic and vaccine-preventable ACSC, while the acute ACSC rates were rather similar. The disparities in annual trends of RDs and SRRs between genders diminished (*p* < 0.0001) from 1996 to 2013 (Table 2).

**Table 2:** Gender disparities that favoured women in ACSC rates in Finland

	SRR [CI 95%]		RD [CI 95%]	
ACSC rates	1996	2013	1996	2013
Vaccine-preventable	2.17 [2.10–2.23]	1.81 [1.77–1.85]	577 [552–602]	492 [472–512]
Chronic	1.55 [1.53–1.57]	1.35 [1.32–1.38]	1227 [1184–1270]	331 [308–353]
Acute	1.22 [1.19–1.25]	1.05 [1.02–1.08]	148 [127–168]	32 [15–48]

[Legend] The decrease in disparities in the annual trends of RDs and SRRs were statistically significant (*p*-value < 0.0001) in every subgroup

*SRR* = standardised rate ratios

*RD* = rate difference (RD)

**Fig. 1:**
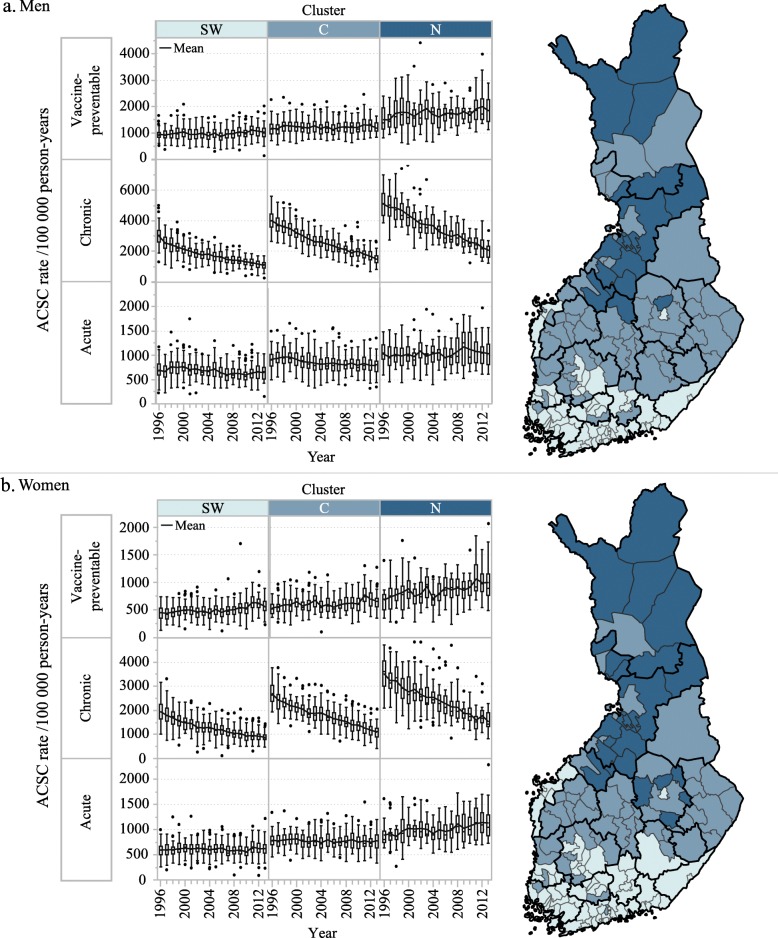
**a** and **b** Allocation of Finnish health centre areas to clusters by their ACSC rates with trajectory modelling. Health centre areas allocated to a single cluster share the similar level and development of age-standardized rates (per 100,000 person-years) of three subgroups of ambulatory care sensitive conditions (ACSCs) in 1996–2013. Box and whisker plots represent the distribution (the median, interquartile range, interquartile range × 1.5 and outliers) of these rates between health centre areas based on each ACSC subgroup, cluster and year. Note the different ranges of y-axis between subgroups and genders. The health centre areas in the map are coloured by the clusters they were allocated to, while thicker black lines mark the borders of hospital districts. SW = southwestern cluster, C = central cluster, N = northern cluster. Adapted and built on the municipality based statistical units, Statistics Finland [32]. The material was downloaded from Statistics Finland’s interface service on 6 October 2017 with the license CC BY 4.0

In trajectory modelling, the applied multifactorial approach consisted of the rates of all three ACSC subgroups separately for both genders. In the model with the best goodness-of-fit, three trajectories (i.e. separate clusters of health centre areas) emerged as health centre areas differed in terms of their levels and rates of change for ACSC rates (Fig. 1 and Additional file 2). The distribution of health centre areas in the trajectories was similar between genders – only slight differences arose. The trajectory with the highest level of ACSC rates comprised 13% of health centre areas for men and 16% for women, mainly in rural parts of northern Finland (i.e. northern cluster). The health centre areas (respectively 42 and 34%) in the central parts of Finland (i.e. central cluster) had a lower level of ACSC rates in every subgroup than those in the northern cluster, and those (45 and 50%) in south-western Finland (i.e. south-western cluster) had the lowest. Further, the northern cluster had 6% of the total Finnish male population and 7% of the female population, while the proportions in the central cluster were 39 and 32%, and in the south-western cluster 55 and 61%, respectively.

In 1996–2013, we observed increasing disparities in annual trends of RDs (*p* ≤ 0.0012) in vaccine-preventable ACSC rates between the northern cluster and both the central and the south-western clusters. The increase of vaccine-preventable ACSC rates in men were: 14% in the northern cluster, 1% in the central cluster and 4% in the south-western cluster in men and respectively 41, 23 and 21% in women. The disparities in annual trends of SRRs in vaccine-preventable ACSC rates increased in men (*p* ≤ 0.0129) but were not significant in women. The main conditions behind this increase were bacterial pneumonia and influenza [data not shown]. Though the disparities in annual trends of RDs decreased between all three clusters in chronic ACSC (*p* < 0.0001), the annual trends in SRRs were similar between clusters (rates increased 62–63% in men and 40–45% in women).

In acute ACSC rates, the annual trends of SRRs and RDs increased in the favour of central and the south-western clusters (*p* ≤ 0.0002 and *p* ≤ 0.0006 respectively): while their rates decreased (16–17% in men and 4–5% in women), those of the northern cluster either decreased less (7% in men) or increased (21% in women). By examining the hospitalisations for individual conditions, we traced this difference to both dental conditions and kidney and urinary tract infections. The increase in these conditions was higher in the northern cluster than elsewhere [data not shown].

Overall, the disparities in RDs between the northern cluster and the other two clusters decreased with chronic ACSC rates but increased with vaccine-preventable and acute ACSC rates. The respective disparities in SRRs remained unchanged in chronic ACSC rates, but increased in vaccine-preventable ACSC rates in men and in acute ACSC rates in both genders. When comparing the central and the south-western clusters, disparities in SRRs and RDs remained rather similar, with the exception of decreasing disparities in RDs in chronic ACSC rates (Table 3).

**Table 3:** Comparison of ambulatory care sensitive condition rates by subgroups and between clusters in Finland

		Vaccine-preventable		Chronic		Acute	
Compared clusters		1996	2013	1996	2013	1996	2013
*Men*							
	SRR	1.61 (1.48–1.74)	1.77 (1.67–1.87)	1.84 (1.77–1.92)	1.91 (1.81–2.01)	1.47 (1.35–1.59)	1.61 (1.50–1.73)
N to SW	Rate	1530/950	1750/990	5210/2820	1980/1030	1030/700	960/590
	RD	580 (460–700)	760 (670–850)	2380 (2190–2580)	940 (840–1040)	330 (250–410)	360 (300–430)
	SRR	1.33 (1.23–1.44)	1.51 (1.43–1.60)	1.29 (1.24–1.35)	1.32 (1.25–1.39)	1.11 (1.02–1.21)	1.25 (1.16–1.34)
N to C	Rate	1530/1150	1750/1160	5210/4020	1980/1500	1030/930	960/770
	RD	380 (260–500)	600 (500–690)	1190 (990–1380)	480 (380–570)	100 (20–190)	190 (120–260)
	SRR	1.21 (1.15–1.26)	1.17 (1.13–1.21)	1.42 (1.39–1.46)	1.45 (1.41–1.50)	1.32 (1.26–1.38)	1.29 (1.25–1.35)
C to SW	Rate	1150/950	1160/990	4020/2820	1500/1030	930/700	770/590
	RD	200 (150–240)	170 (130–200)	1200 (1120–1270)	470 (430–500)	220 (190–260)	170 (150–200)
*Women*							
	SRR	1.52 (1.41–1.64)	1.76 (1.67–1.87)	1.92 (1.86–1.99)	1.83 (1.75–1.91)	1.48 (1.39–1.58)	1.89 (1.78–2.00)
N to SW	Rate	690/450	970/550	3520/1830	1490/820	860/580	1050/550
	RD	240 (190–290)	420 (370–470)	1690 (1580–1800)	680 (610–740)	280 (230–340)	490 (430–550)
	SRR	1.31 (1.21–1.41)	1.50 (1.41–1.59)	1.30 (1.26–1.35)	1.39 (1.33–1.46)	1.10 (1.03–1.17)	1.38 (1.30–1.47)
N to C	Rate	690/530	970/650	3520/2710	1490/1070	860/790	1050/760
	RD	160 (110–210)	320 (270–380)	820 (700–930)	420 (350–490)	80 (20–130)	290 (230–350)
	SRR	1.16 (1.11–1.22)	1.18 (1.14–1.22)	1.48 (1.45–1.51)	1.31 (1.27–1.35)	1.35 (1.30–1.41)	1.37 (1.32–1.42)
C to SW	Rate	530/450	650/550	2710/1830	1070/820	790/580	760/550
	RD	70 (50–100)	100 (80–120)	870 (820–930)	250 (230–280)	210 (180–230)	200 (180–230)

The increase in disparities in annual trends of RDs was significant between the northern cluster and the other two clusters in vaccine-preventable ACSC rates and acute ACSC rates. In chronic ACSC rates the decrease of these disparities was significant between all three clusters. The increase in disparities in annual trends of SRRs was significant between the northern cluster and both of the two other clusters in vaccine-preventable ASCS rates in men and acute ACSC rates in both genders. The ACSC rates presented were calculated from the total population of each of the clusters and thus differ from the mean rates of health centre areas presented in Additional file 2

*SW* = southwestern cluster

*C* = central cluster

*N* = northern cluster

*SRR* = standardised rate ratios (and their 95% clearance intervals)

Rate = age-standardized rates/100,000 person-years

*RD* = rate differences (and their 95% clearance intervals)

All the descriptive area-level factors correlated quite strongly with each other. When estimated using GEE, the factors that significantly associated with the distribution of health centre areas in the trajectories for both genders were as follows: increase in morbidity index (*p* ≤ 0.033), number of inpatient PHC periods (*p* ≤ 0.0002) and number of pensioner’s care allowance of recipients aged ≥65 (*p* ≤ 0.0038) associated with the higher likelihood that health centre area was allocated to cluster with higher ACSC rates (i.e. northern cluster). Further, increase in proportion of both inhabitants aged ≥65 (*p* < 0.0001) and with tertiary education (*p* ≤ 0.0381) and proportion of inhabitants reimbursed for private health care use (*p* ≤ 0.0203) associated with the higher likelihood that health centre area was allocated to cluster with lower ACSC rates (i.e. southwestern cluster). Two factors were significant for women only: the increase in number of reimbursed private dental care visits (*p* = 0.0005) associated with the higher likelihood that health centre area was allocated to cluster with higher ACSC rates and the increase in municipal degree of urbanisation (*p* = 0.0295) associated with the higher likelihood that health centre area was allocated to cluster with lower ACSC rates. No other area-level factors improved the model. The health centre areas in the northern cluster were characterised by low SEP, less usage of private health and dental care, and a lower degree of urbanisation, as well as high morbidity, limitations in ADL among the elderly and a greater number of inpatient PHC periods (Table 4). The findings were the opposite for the south-western cluster. The central cluster had the highest proportion of inhabitants aged ≥65.

**Table 4:** Characteristics of area-level factors in health centre areas by clusters in 1996–2013 (mean ± SE)

	Men			Women		
Clusters	SW	C	N	SW	C	N
Health centre areas (n)	*59*	*55*	*17*	*65*	*45*	*21*
Healthcare-dependant factors						
Inpatient primary health care periods per 1000 inhabitants	30 ± 0.5	70 ± 1.1	98 ± 2.4	43 ± 0.7	92 ± 1.4	114 ± 2.5
Reimbursed private dental care visits per 1000 inhabitants	460 ± 6	290 ± 5	210 ± 6	450 ± 6	270 ± 6	210 ± 6
Proportion of people reimbursed for private health care use (%)	23 ± 0.1	17 ± 0.1	13 ± 0.2	37 ± 0.2	31 ± 0.2	23 ± 0.2
Populations sociodemographic characteristics						
Proportion of population aged ≥65 (%)	21 ± 0.1	24 ± 0.1	21 ± 0.3	21 ± 0.1	24 ± 0.2	22 ± 0.2
Proportion of population with tertiary education (%)	24 ± 0.2	18 ± 0.2	17 ± 0.3	23 ± 0.2	18 ± 0.2	17 ± 0.3
Municipal characteristics						
Degree of urbanization (%)	80 ± 0.4	70 ± 0.4	57 ± 0.7	80 ± 0.5	70 ± 0.4	65 ± 0.8
Other health-related factors						
Morbidity index	94 ± 0.4	112 ± 0.6	120 ± 0.9	95 ± 0.4	112 ± 0.7	120 ± 0.9
Pensioner’s care allowance of recipients aged ≥65 per 1000 inhabitants of the same age	108 ± 1	144 ± 1	169 ± 2	171 ± 2	221 ± 2	248 ± 2

All the differences between group averages (tested with ANOVA) were statistically significant (*p* < 0.0001) for every area-level factor

*SW* = southwestern cluster

*C* = central cluster

*N* = northern cluster

## Discussion

We observed the geographical distribution of ACSC rates and their development over time in Finnish health centre areas in the years 1996–2013. Further, we differentiated three clusters of health centre areas using trajectories based on the level and development of ACSC rates of these areas and assessed whether the chosen area-level factors would describe this distribution in specific trajectories. Our findings illustrate increasing absolute and relative geographical disparities in vaccine-preventable and acute ACSC rates: the rates in the northern cluster were constantly the highest, but they also increased over time unlike in the other areas. This partially supports the suspected deterioration in PHC. Relative disparities in chronic ACSC rates remained unaffected, though the rates and absolute disparities decreased by almost two-thirds within the study period. The health centre areas in the northern cluster consistently had the highest level of ACSC rates, but their inhabitants were the least educated and had the highest coverage of morbidity. Further, these areas had the highest proportion of elderly people with limitations in ADL and the highest usage of PHC inpatient wards, but the lowest usage of private health care and private dental care. In all these characteristics, the south-western cluster had the opposite. These findings describe the development of Finnish PHC in a surprisingly plausible manner: PHC in rural municipalities (where the need for care seems to be higher than elsewhere) was lagging behind developments in other parts of the country. This calls for further investments and novel solutions in the provision of rural PHC services.

### Strengths and weaknesses of the study

This observational study described the geographical distribution and development over time of ACSC rates in Finland, which has not been previously studied. In comparison to previous studies assessing the geographical distribution of ACSC rates within other European countries, we assessed the rates of all three ACSC subgroups in the same model, which enabled a more comprehensive approach. However, the applied model is a grouping tool, which averages the characteristics of the several health centre areas observed. Also, there might exist health centre areas at the border of trajectories, whose grouping might change by just a slight change in ACSC rates. Thus, the evaluation of individual health centre areas or the intra-cluster variation among them requires further studies with more specific methods. This involves also the use of our results for direct evaluation of health centres, as ACSC rates can also be affected by factors other than the quality of PHC. Thus, the results need to be interpreted with caution. As we could not study associations between the explanatory factors and the ACSC hospitalisations at the individual level, we had to rely on descriptive analysis and cannot assess the causality between the area-level factors and ACSC rates. Thus, we cannot completely rule out the possibility of ecological fallacy. However, we calculated ACSC rates over a comprehensive time frame from individual hospitalisation data of Finnish registers, which have been shown to be of good quality [33]. As the included diagnose of unspecified pneumonia (J18.9) captures also hospitalisations that are unavoidable by currently available vaccinations, the vaccine-preventable ACSC rates needs to be interpreted with caution. With community-acquired pneumonias in the Finnish elderly *Streptococcus pneumoniae* is included in at least fifth of the radiologically-confirmed cases and influenza A approximately in a tenth [34]. Almost in the half of these cases pathogen remains unidentified. As the proportion of unspecified pneumonia in our hospitalisation data was larger than this, we interpreted that part of them were likely to be unrecognized pneumococcal diseases or influenza – an effect which scale we could not however ascertain. Further, we had to limit our analysis to population aged ≥20 as we had no sociodemographic data for population under 20 years old. However, we assume this had only minor effect on the geographic distribution of ACSC rates as the majority of ACSC conditions occur in the elderly [21].

### Comparison with the literature

Finland’s decreasing trends in total ACSC rates are similar to those of Canada [35] and Denmark [36], but contrary to increasing trends in France [37], Sweden [38], and the UK [39]. However, direct comparison of the results from different studies and countries is not feasible since the definition of ACSC varies. We found only a few studies that applied a similar definition of ACSC between countries and within the same statistical model [40, 41]. Even these studies, though, mainly analyse chronic conditions in order to retain comparability and avoid difficulties in interpreting the effect of different hospitalisation practices. Further, the elevated level of ACSC rates in rural areas is consistent with findings from Germany and Canada [42, 43], but it contradicts findings from Spain, where the distance to hospitals appears to decrease ACSC rates [44].

### Possible explanations for differences between clusters

Finland’s slight increase in vaccine-preventable ACSC rates occurred due to bacterial pneumonia and influenza. This is in line with previous studies reporting that other causes for vaccine-preventable ACSC have mostly disappeared in Finland due to high vaccination prevalence [45]. This applies also to hepatitis A and B, the incidence of which is quite low even though the vaccinations are only offered to high-risk groups [46, 47]. As the incidence of both bacterial pneumonia and influenza are high [34, 48, 49], increasing the currently low vaccination coverages [50, 51] could reduce such ACSC hospitalisations. The only data we had on the geographical distribution of vaccine coverage for these two diseases was for influenza among small children and the elderly in 2013, and thus we did not include these in the analysis. While co-morbidities and old age predispose people [52, 53] to pneumonias, it is likely that these risk factors contributed to the geographic disparities we observed. In 2010, Finland implemented a national infant vaccination program with a 10-valent pneumococcal conjugate vaccine (PCV10), and it has since been shown to provide herd protection and decrease hospitalisations for both pneumococcal and all-cause pneumonias [51]. Thus, it is possible that the increase in vaccine-preventable ASCS that we observed will either stagnate or begin to decrease after 2013.

It is likely that the decrease in chronic ACSC rates occurred due to nationwide improvements in knowledge, screening, treatment, and follow-ups on chronic diseases [54–56]. This interpretation emphasises that different local approaches of PHC in Finland seem to play only a minor role in decreasing chronic ACSC rates, which is consistent with previous studies that access to PHC is not the main factor affecting ACSCs or its geographical distribution [18, 57]. Though these findings support the suspected deterioration in PHC, part of the increase in relative disparities might have arisen from the different care pathways and hospitalisation criteria used in various Finnish hospital districts. The trajectories abided by the geographical borders of these districts: each district included health centre areas only in two consecutive clusters, with the exception of a single district in eastern Finland. Also, we assume that the differences in levels of SEP and the morbidity of inhabitants in health centre areas maintained the disparities among the different trajectories, but further research on this is needed.

The increase in acute ACSC rates in the northern cluster occurred due to hospitalisations for kidney and urinary tract infection (UTI) and dental conditions. Our finding on UTI hospitalisations is similar to that regarding the elderly in the UK [39], where it has been estimated that almost half of such admissions are incorrectly diagnosed [58]. One suggested reason for this inaccuracy, the usage of UTI as an acceptable cause for hospitalisations of frail older adults with an uncertain diagnosis [58], should also be considered in Finland. Thus, the increase in UTI hospitalisations likely reflects the accumulation of risk factors, such as an ageing population, diabetes and obesity [59, 60]. Further, the limitations of ADL might mediate the link between UTIs [61] in the elderly and increased ACSC rates [23] through delays in accessing health centres. Our findings were in line with this possible interpretation: while the health centre areas in the central cluster had the largest proportion of elderly inhabitants, those in the northern cluster suffered more often from limitations in ADL. The hospitalisations for dental conditions in the northern cluster began to increase in our data in 2002, when Finland implemented an oral health care reform that removed age limitations on both access to Public Dental Services (PDS) and reimbursements for private dental care [62]. This reform increased the demand of PDS [62], but not of the private dentists [63]. It is likely that the observed geographic disparities resulted both from a previously unmet need for dental care by adults unable to access the private services at their own expense in the northern cluster and from a deterioration in performance of a since overburdened PDS. Since the reform, PDS has received plenty of new working-aged and elderly users [64], mainly of low SEP and with a relatively high need for care [65, 66]. Unsurprisingly, the initial state of oral health of PDS patients has been poorer after the reform [66]. There still exists an unmet need for oral health care that has a clear socioeconomic gradient: the risk of being a non-user increases with lower incomes [67].

For area-level factors, our findings supported the previously documented effect of both morbidity and SEP on ACSC rates: the health centre areas with high morbidity and low SEP also had a high level of ACSC rates [18, 22, 23]. The high usage of private health care providers, on the contrary, occurred in areas with low morbidity and high SEP. In Finland, these providers offer an alternative route to both GP and secondary care outpatient consultations, but they are located mainly in urban areas. As the reimbursements cover only a fraction of the consultation fee, this route benefits more those with higher SEP – thus, this factor appears to be an indirect SEP indicator. The disparities in the usage of PHC inpatient wards might relate to a reduction in the number of beds in elderly homes [68], leaving PHC no other options than to hospitalise those who would need only institutional social care. This assumption could be further supported by the high morbidity and limitations in ADL among the elderly in northern Finland as well as the previously mentioned possibility of inaccurate diagnostics for UTIs. One possibility is that the cost savings of institutional social care in recent decades have been partially translated into health care expenditures. However, further research on this is needed.

### Implications for clinicians and policy makers

Our findings relate less to differences in local PHC health centres and more to bigger health policy decisions affecting nationwide developments. We observed increasing relative disparities that partly supported the hypothesised deterioration in PHC in Finland due to a lack of national stewardship and decentralization – these geographic disparities appeared to follow an uneven distribution of population characteristics for SEP and morbidity. Wilding et al. [69] estimate that those persons with a limiting long-term illness are less likely to move longer distances, and when doing so prefer rural or highly urbanised areas. Thus, the development of population characteristics in rural Finland is unlikely to change in the near future. This highlights the urgent need for novel solutions in providing PHC services to rural areas: the question of how to address selective depopulation in rural Finland and elevated health and social care needs among inhabitants in those areas remains unanswered. With further analysis it might be possible to apply ACSC rates as a tool to identify both well and poorly performing health centres – and to promote transfer of well-functioning care pathways and protocols between them.

### Unanswered questions and future research

Due to the lack of individual data on SEP and co-morbidities, we were unable to give any estimates of their effect on geographical disparities in ACSC rates. Future studies need also to account for possible geographic disparities in ACSC rates of youth populations. Also, we were unable to provide any insights into on-going debates on the usage of ACSC rates as a PHC quality indicator – which necessitates data that follows patients across the boundaries of different health care systems and through care episodes for specified diseases. The possible association of elevated ACSC rates and a high usage of PHC inpatient wards should also be examined in further detail. Though we observed gender and geographical disparities for ACSC rates, we were unable to assess possible ethnic inequities. More research on this is needed in Finland, as there exists minorities whose need for care very likely exceeds those of the majority population [70].

## Conclusions

The performance of PHC in Finland seemingly developed rather well between 1996 and 2013, mainly due to a substantial reduction in the number of hospitalisations for chronic ACSC conditions. Relative geographic disparities mainly increased, partly supporting the hypothesised slow deterioration in PHC quality. The accumulation of health problems associated with an ageing population appeared to burden rural areas, highlighting the need for both further investments and novel solutions in the provision of health care services. The trajectory analysis of rates of the three ACSC subgroups over time provided a broad and plausible picture on the development of PHC in Finland, which could still be refined with individual data on morbidity and socio-economic position.

## Additional files

Additional file 1: The list of ACSC conditions with definition notes. (PDF 130 kb)

Additional file 2: Mean ACSC rates in health centre areas and estimates of the multi-trajectory model. (PDF 244 kb)

Additional file 3: The original protocol for the study. (PDF 92 kb)

## Data Availability

The data that support the findings of this study are available from the National Institute for Health and Welfare (THL), but restrictions apply to the availability of these data, which were used under license for the current study, and so are not publicly available. Data are however available from the authors upon reasonable request and with permission of THL.

## Abbreviations

ACSC: Ambulatory care sensitive conditions
ADL: Activities of daily living
GBTM: Group-based trajectory modelling
PDS: Public Dental Services
PHC: Primary health care
SEP: Socioeconomic position
THL: National Institute for Health and Welfare, Finland
UTI: Kidney and urinary tract infection
