# Supplementary material for: Trajectory modelling of ambulatory care sensitive conditions in Finland in 1996–2013: assessing the development of equity in primary health care through clustering of geographic areas – an observational retrospective study
Source: BMC Health Serv Res. 2019 Sep 4;19:629. doi: 10.1186/s12913-019-4449-7 (PMC6727548; doi:10.1186/s12913-019-4449-7)

Additional file 2.

Mean ambulatory care sensitive condition (ACSC) rates in health centre areas (n=131) in Finland in 1996-2013.

Men

Year	Vaccine-preventable	Chronic	Acute
	mean [min, max]	mean [min, max]	mean [min, max]
1996	1115 [516, 2395]	3723 [1311, 7029]	831 [224, 1497]
1997	1088 [370, 2343]	3377 [1127, 6681]	592 [223, 1522]
1998	1195 [529, 3071]	3236 [881, 7079]	595 [327, 1479]
1999	1211 [497, 3114]	3116 [1386, 7430]	615 [338, 1659]
2000	1217 [530, 3201]	2895 [992, 7651]	624 [327, 1551]
2001	1148 [425, 3529]	2728 [806, 6164]	612 [204, 1751]
2002	1149 [466, 4417]	2524 [900, 6081]	619 [225, 1505]
2003	1229 [455, 2944]	2394 [988, 6709]	622 [322, 1948]
2004	1110 [449, 2833]	2395 [1081, 4894]	591 [386, 1846]
2005	1170 [296, 2538]	2218 [605, 4027]	614 [331, 1502]
2006	1083 [370, 2891]	2140 [616, 4624]	630 [331, 1568]
2007	1164 [398, 2582]	1976 [550, 4262]	571 [325, 1744]
2008	1173 [423, 2403]	1932 [703, 4318]	578 [388, 1658]
2009	1203 [348, 2322]	1800 [866, 4092]	588 [345, 1841]
2010	1175 [522, 2705]	1775 [396, 3906]	553 [350, 1801]
2011	1290 [572, 3105]	1663 [484, 3469]	650 [285, 1549]
2012	1276 [614, 3985]	1521 [394, 4007]	613 [289, 1976]
2013	1199 [132, 2892]	1388 [249, 3365]	616 [151, 1564]

Women

Year	Vaccine-preventable	Chronic	Acute
	mean [min, max]	mean [min, max]	mean [min, max]
1996	515 [128, 1402]	2461 [1005, 4715]	708 [259, 1617]
1997	525 [224, 1407]	2245 [763, 4585]	710 [200, 1534]
1998	555 [222, 1120]	2156 [797, 4425]	709 [270, 1374]
1999	574 [190, 1767]	1994 [558, 4026]	741 [301, 1660]
2000	612 [153, 1383]	1911 [1031, 4038]	756 [305, 1442]
2001	562 [270, 1447]	1884 [813, 4836]	733 [237, 1623]
2002	565 [228, 1264]	1713 [269, 4832]	734 [337, 1594]
2003	617 [229, 1256]	1684 [420, 3828]	742 [329, 1578]
2004	524 [97, 1106]	1701 [723, 4546]	699 [222, 1297]
2005	586 [272, 1138]	1591 [439, 4186]	741 [259, 1435]
2006	560 [116, 1335]	1525 [120, 4013]	725 [323, 1457]
2007	589 [238, 1170]	1435 [454, 4699]	704 [373, 1360]
2008	605 [273, 1318]	1386 [589, 3763]	735 [98, 1733]
2009	620 [206, 1709]	1299 [506, 2800]	734 [247, 1566]
2010	617 [158, 1591]	1240 [515, 2752]	705 [314, 1557]
2011	749 [288, 1856]	1164 [312, 3410]	771 [332, 1625]
2012	708 [346, 1600]	1151 [494, 3105]	749 [89, 1703]
2013	667 [229, 2075]	1056 [406, 2279]	758 [233, 2292]

Mean vaccine-preventable ACSC rates in men by clusters of health centre areas in 1996-2013. SW = southwestern cluster, C = central cluster, N = northern cluster

Vaccine-preventable

Year	SW (n=59)	C (n=55)	N (n=17)
	mean [min, max]	mean [min, max]	mean [min, max]
1996	949 [516, 1660]	1173 [702, 2262]	1501 [795, 2395]
1997	918 [370, 1360]	1146 [554, 1724]	1490 [942, 2343]
1998	954 [529, 1660]	1285 [712, 2348]	1738 [1003, 3071]
1999	996 [497, 1843]	1264 [660, 2130]	1782 [613, 3114]
2000	1035 [530, 2088]	1242 [607, 1893]	1766 [564, 3201]
2001	940 [425, 1545]	1220 [697, 2085]	1632 [739, 3529]
2002	938 [466, 1594]	1179 [756, 1663]	1788 [928, 4417]
2003	993 [455, 1623]	1269 [753, 2067]	1922 [666, 2944]
2004	887 [449, 1567]	1166 [762, 1889]	1697 [648, 2833]
2005	985 [296, 1616]	1234 [692, 2183]	1602 [943, 2538]
2006	857 [370, 1371]	1123 [718, 1805]	1736 [1091, 2891]
2007	956 [398, 1520]	1206 [753, 1769]	1750 [1195, 2582]
2008	971 [423, 1655]	1231 [675, 2007]	1689 [921, 2403]
2009	1036 [348, 1762]	1199 [622, 1737]	1799 [1363, 2322]
2010	999 [522, 1628]	1207 [760, 1917]	1686 [1158, 2705]
2011	1100 [572, 1611]	1302 [741, 2460]	1913 [1028, 3105]
2012	1060 [614, 1800]	1285 [810, 1890]	1995 [1351, 3985]
2013	1025 [132, 2020]	1190 [832, 1643]	1831 [1118, 2892]

Chronic

Year	SW (n=59)	C (n=55)	N (n=17)
	mean [min, max]	mean [min, max]	mean [min, max]
1996	3031 [1311, 5018]	4034 [2669, 5633]	5119 [3843, 7029]
1997	2568 [1127, 3724]	3779 [2555, 5208]	4886 [2757, 6681]
1998	2427 [881, 3478]	3602 [2521, 5195]	4859 [3582, 7079]
1999	2295 [1386, 3925]	3510 [2339, 5094]	4691 [3593, 7430]
2000	2133 [992, 3104]	3247 [2204, 4723]	4400 [3118, 7651]
2001	2032 [806, 3179]	3046 [1837, 4198]	4118 [2214, 6164]
2002	1868 [900, 2920]	2828 [1671, 3951]	3813 [2878, 6081]
2003	1771 [988, 2739]	2644 [1734, 4035]	3747 [2094, 6709]
2004	1830 [1081, 2641]	2599 [1564, 3679]	3697 [2414, 4894]
2005	1655 [605, 2923]	2490 [1620, 3308]	3292 [2427, 4027]
2006	1646 [616, 2523]	2367 [1439, 3521]	3118 [2261, 4624]
2007	1465 [550, 2274]	2214 [1322, 3193]	2982 [2211, 4262]
2008	1425 [703, 2353]	2116 [1242, 3686]	3102 [2379, 4318]
2009	1409 [866, 2344]	1916 [1089, 2974]	2784 [1868, 4092]
2010	1315 [396, 1908]	2014 [1035, 3906]	2594 [1243, 3495]
2011	1262 [484, 1851]	1825 [1190, 2722]	2535 [1799, 3469]
2012	1162 [394, 1723]	1683 [664, 2678]	2245 [1338, 4007]
2013	1082 [249, 1689]	1507 [802, 2675]	2067 [1211, 3365]

Acute

Year	SW (n=59)	C (n=55)	N (n=17)
	mean [min, max]	mean [min, max]	mean [min, max]
1996	695 [224, 1175]	915 [492, 1497]	1030 [589, 1490]
1997	663 [223, 1016]	937 [549, 1515]	966 [453, 1522]
1998	763 [327, 1479]	965 [457, 1410]	1016 [709, 1436]
1999	748 [338, 1296]	962 [657, 1659]	984 [441, 1626]
2000	768 [327, 1216]	920 [525, 1551]	1023 [721, 1388]
2001	706 [204, 1751]	868 [458, 1449]	970 [507, 1734]
2002	720 [225, 1114]	858 [363, 1454]	1085 [475, 1505]
2003	683 [413, 1027]	839 [322, 1496]	980 [598, 1948]
2004	681 [389, 1008]	813 [386, 1160]	1029 [400, 1846]
2005	712 [331, 1301]	826 [443, 1262]	1053 [692, 1502]
2006	641 [331, 940]	840 [484, 1568]	953 [493, 1389]
2007	598 [325, 1037]	815 [398, 1449]	994 [626, 1744]
2008	629 [391, 1203]	802 [388, 1198]	1081 [641, 1658]
2009	622 [345, 1072]	836 [572, 1289]	1172 [528, 1841]
2010	596 [350, 1003]	794 [483, 1167]	1119 [660, 1801]
2011	636 [285, 1226]	828 [486, 1309]	1079 [683, 1549]
2012	656 [289, 1050]	794 [318, 1326]	1059 [601, 1976]
2013	640 [151, 1025]	786 [347, 1356]	1033 [602, 1564]

Mean vaccine-preventable ACSC rates in women by clusters of health centre areas in 1996-2013. SW = southwestern cluster, C = central cluster, N = northern cluster

Vaccine-preventable

Year	SW (n=66)	C (n=45)	N (n=22)
	mean [min, max]	mean [min, max]	mean [min, max]
1996	445 [128, 740]	531 [361, 793]	712 [351, 1402]
1997	433 [224, 742]	557 [275, 970]	761 [276, 1407]
1998	455 [222, 719]	591 [261, 1019]	791 [276, 1120]
1999	475 [190, 798]	595 [222, 1047]	862 [348, 1767]
2000	493 [153, 839]	648 [396, 1027]	907 [531, 1383]
2001	492 [270, 797]	570 [303, 867]	780 [284, 1447]
2002	458 [228, 916]	610 [325, 1186]	812 [451, 1264]
2003	474 [229, 718]	674 [413, 1090]	939 [547, 1256]
2004	436 [97, 710]	561 [97, 906]	720 [285, 1106]
2005	506 [272, 864]	598 [346, 1138]	814 [405, 1138]
2006	450 [116, 730]	549 [358, 846]	927 [505, 1335]
2007	491 [238, 1067]	595 [349, 885]	879 [238, 1170]
2008	498 [273, 790]	617 [398, 984]	919 [424, 1318]
2009	540 [206, 1709]	618 [250, 965]	897 [458, 1709]
2010	522 [158, 891]	609 [278, 1002]	943 [501, 1591]
2011	629 [288, 1002]	770 [459, 1306]	1099 [687, 1856]
2012	620 [346, 1199]	694 [380, 1027]	1021 [545, 1600]
2013	562 [229, 892]	657 [351, 1277]	1048 [482, 2075]

Chronic

Year	SW (n=66)	C (n=45)	N (n=22)
	mean [min, max]	mean [min, max]	mean [min, max]
1996	1933 [1005, 3161]	2705 [1936, 3756]	3579 [2090, 4715]
1997	1780 [763, 3309]	2429 [1440, 3770]	3300 [2106, 4585]
1998	1677 [797, 2539]	2329 [1678, 3360]	3282 [1478, 4425]
1999	1537 [558, 2346]	2194 [1358, 3048]	2985 [1761, 4026]
2000	1472 [1031, 2330]	2137 [1625, 3326]	2825 [1230, 4038]
2001	1447 [813, 2391]	2041 [1557, 3020]	2959 [1535, 4836]
2002	1277 [269, 2124]	1882 [1121, 3066]	2752 [1298, 4832]
2003	1260 [420, 2055]	1878 [1270, 3082]	2596 [1620, 3828]
2004	1285 [723, 2284]	1895 [1009, 3035]	2635 [1385, 4546]
2005	1187 [439, 1947]	1773 [1014, 2798]	2493 [1354, 4186]
2006	1172 [120, 2004]	1660 [812, 2653]	2356 [1482, 4013]
2007	1085 [454, 1869]	1596 [725, 2239]	2262 [849, 4699]
2008	1041 [589, 1894]	1533 [865, 2603]	2195 [1519, 3763]
2009	1032 [506, 2052]	1401 [799, 2367]	1921 [1230, 2800]
2010	931 [515, 1639]	1395 [767, 2257]	1888 [1168, 2752]
2011	946 [312, 1658]	1266 [534, 2161]	1672 [793, 3410]
2012	921 [494, 1681]	1181 [650, 2042]	1839 [1074, 3105]
2013	873 [406, 1440]	1092 [406, 2053]	1559 [820, 2279]

Acute

Year	SW (n=66)	C (n=45)	N (n=22)
	mean [min, max]	mean [min, max]	mean [min, max]
1996	590 [259, 1000]	789 [457, 1344]	922 [639, 1617]
1997	592 [200, 919]	776 [525, 1136]	946 [543, 1534]
1998	595 [270, 1254]	796 [482, 1374]	886 [270, 1374]
1999	615 [301, 1103]	807 [391, 1070]	1003 [409, 1660]
2000	624 [305, 835]	814 [519, 1220]	1045 [681, 1442]
2001	612 [237, 1267]	769 [423, 995]	1039 [785, 1623]
2002	619 [337, 911]	759 [467, 1141]	1049 [624, 1594]
2003	622 [329, 994]	784 [422, 1194]	1031 [734, 1578]
2004	591 [222, 987]	741 [384, 1192]	942 [536, 1297]
2005	614 [259, 918]	796 [544, 1324]	1016 [612, 1435]
2006	630 [323, 1060]	743 [450, 1118]	987 [557, 1457]
2007	571 [373, 907]	758 [444, 1059]	1007 [554, 1360]
2008	578 [98, 904]	774 [372, 1279]	1142 [615, 1733]
2009	588 [247, 926]	801 [517, 1254]	1050 [636, 1566]
2010	553 [314, 857]	748 [371, 1181]	1096 [733, 1557]
2011	650 [332, 1069]	775 [386, 1550]	1143 [701, 1625]
2012	613 [89, 988]	750 [448, 1153]	1167 [701, 1703]
2013	616 [233, 1222]	781 [338, 1198]	1184 [723, 2292]

Estimates (uniform line) and 95% confidence intervals (dashed line) of the trajectory model with the three clusters of health centre areas and three subgroups of ACSC in both genders.

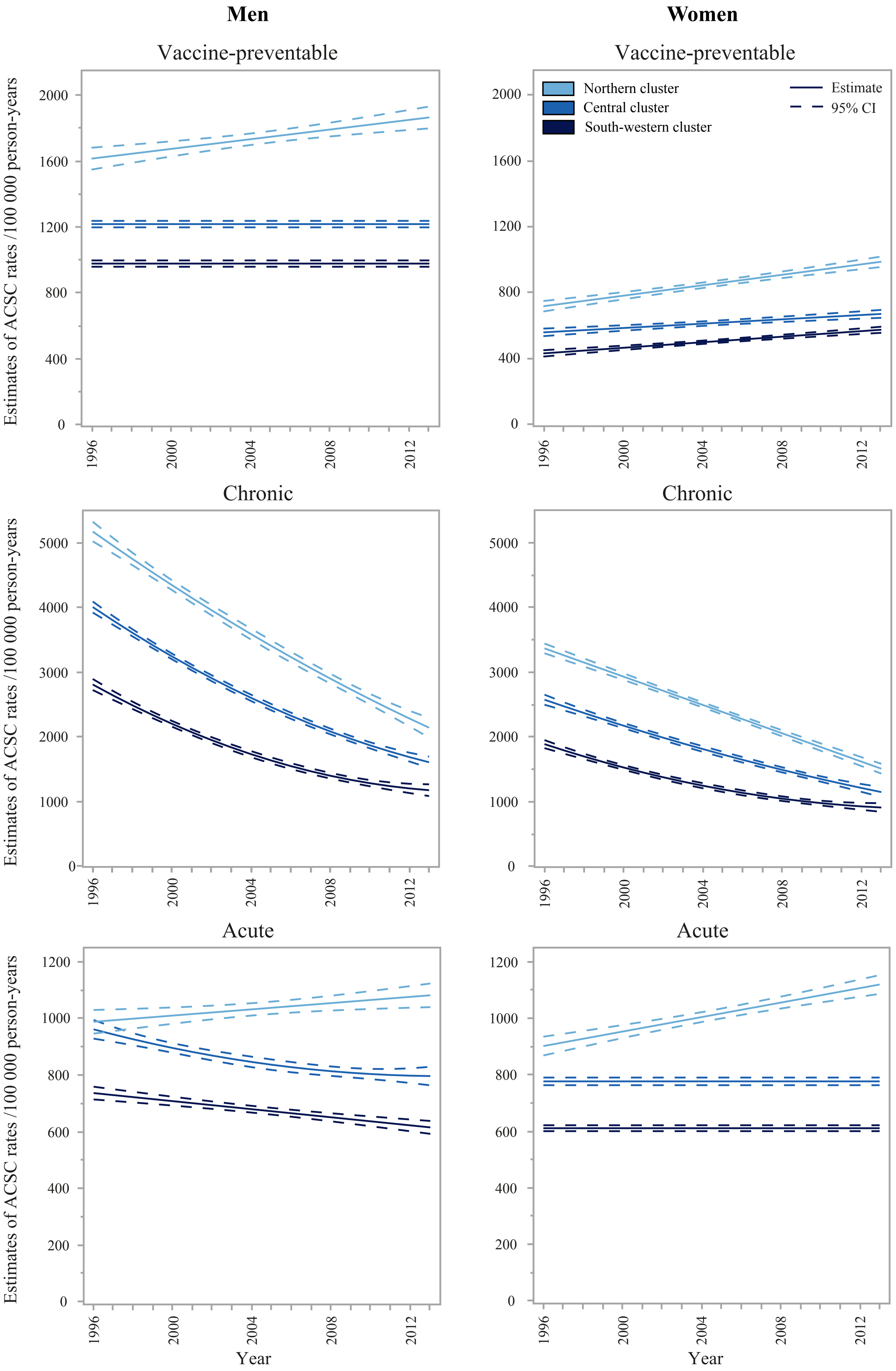

Supplement: Supplementary file 2 — Mean ACSC rates in health centre areas and estimates of the multi-trajectory model. (PDF 244 kb) [file 12913_2019_4449_MOESM2_ESM.pdf]
