# Supplementary material for: Trajectory modelling of ambulatory care sensitive conditions in Finland in 1996–2013: assessing the development of equity in primary health care through clustering of geographic areas – an observational retrospective study
Source: BMC Health Serv Res. 2019 Sep 4;19:629. doi: 10.1186/s12913-019-4449-7 (PMC6727548; doi:10.1186/s12913-019-4449-7)
Supplement: Supplementary file 3 — The original protocol for the study. (PDF 92 kb) [file 12913_2019_4449_MOESM3_ESM.pdf]

Effect of spatial social polarisation on geographical variation of potentially preventable hospitalizations in Finland in 1996-2013: an evaluation of health system performance over time

Operative protocol: 28.9.2016, Markku Satokangas

Background

Potentially preventable hospitalizations (PPH), also referred as acute care sensitive conditions (ACSCs), are a selection of medical conditions in which hospital admission might be avoided with proper treatment in primary health care (PHC). [1] In the United States ACSC serves as an indicator of access to primary care, while in Europe - with PHC of universal access - it is suggested as an indicator of quality of PHC. Such usages necessitate risk adjustment for both population health, e.g. patient morbidity, disease prevalence, and socioeconomic status. [2, 3] While the main body of the research evaluating the association of PPH and PHC access originates from US, the evidence suggests inverse association to a lesser extent also in the European health care systems. [4, 5]

Criteria for PPH and organization of health care vary between countries, which makes international comparison difficult. Additionally, previous studies about geographic variation in Europe and Australia yield contradictory results. In Madrid, Spain, the PPH of elderly accumulates into the city periphery. [6] However, in the rural areas of Spain larger proportion of elderly and longer distance from the hospital decreases the PPH. [7] One study in Switzerland found no significant association between physician density and PPH [3] , while another states that the high density of PHC physicians decreases PPH and the higher density of outpatient specialists increases it. [8] In Germany increase in rurality and number of hospital beds increases PPH, while specialist density decreases it. [9] However, findings from Australia and Spain suggest that socioeconomic factors have a considerably bigger effect on the level of PPH than primary care physician density. [10, 11] Though the physician density appears to be only a minor explanatory factor, increased physician workload, measured with the number of consultations, seems to increase the occurrence of PPH. [11, 12]

Also the effect of several other, mostly area-level factors have been assessed: 1) health care dependant variables: number of hospital beds, 2) characteristics of the area of residence: rurality, unemployment, 3) sociodemographic variables: age, sex, income, and 4) individual health characteristics: life expectancy, comorbidities.

Context in Finland

Finland sustains a tax-funded health care system that provides PHC with universal access. The current municipal responsibility for organizing primary health care dates

back to Primary Health Care Act in 1972. This law obliged each municipality to establish a local health centre, leading to a countrywide PHC network. With a strong guidance of the state the geographical equity of health centres was upheld until the abolition of the Social and Health Board at the end of 1992. [13] In 1993, to strengthen the municipal autonomy the state's economic control over social and health services of municipalities was removed. As opposed to earlier, state institutions role changed to only instructional, without any possibility of sanctioning municipalities with too low service level. [14] Simultaneously due to the recession the amount of government grants for PHC decreased.

Since 1990s the decentralized PHC has shown signs of slow deterioration when compared to both special, and private health care. Between 2000 and 2010 while the total physician workforce increased 24%, in public health centres it decreased 3%. [13] Also number of physician appointments in PHC have decreased annually, while in special, and occupational health care these visits have vastly increased. [15] Though this can be explained with more efficient ways to organise care, for example other occupational groups treating less challenging consultations, it has also resulted in increased GP workload. It would be surprising if this development, with the current level of municipal freedom in PHC organizing, would not present itself as geographical polarization between municipalities.

No previous research exists about geographical variation of PPH in Finland. No previous group-based trajectory model analysis about geographical variation of PPH and characteristics of poorly performing municipalities exists.

In this study we 1) assess the geographical variation of PPH in Finnish municipalities in 1996-2013, 2) group the municipalities according to their PPH over time with group-based trajectory models, and 3) analyse if area-level healthcare-dependant variables explain any differences between the poorly and well-performing groups. Our hypothesis was, that increasing or stagnant development of municipal PPH was associated with reduced local PHC resourcing and increasing physician workload.

Methods

We divided Finland into small areas, each representing a district served by a single primary health care provider unit, i.e. health centre or station. Finnish health centres serve either a single municipality or a consolidation of tiny municipalities, and in cities health centres are divided into health stations. In 2008 rural health centres covered areas with population of 3056-38151, and urban health stations covered areas with population of 3255-39723.

For these small areas the HILMO-register of National Institute for Health and Welfare yielded the number of PPH between 1996 and 2013. As definition of PPH we applied a list used by National Health Service in UK (representing episodes potentially preventable by outpatient care). [16] Conditions included were angina, asthma, bacterial pneumonia, COPD, cellulitis, congestive heart failure, convulsions, dehydration, dental conditions, diabetes complications, epilepsy, gastroenteritis, hypertension, immunization-related and preventable conditions, iron deficiency anemia, kidney and urinary tract infections, nutritional deficiencies, pelvic inflammatory disease, perforated or bleeding ulcer, and severe ENT infections. These conditions were then categorized into acute, chronic and vaccine-preventable causes according to the Atlas of Avoidable Hospitalisations in Australia. [17] This categorization is suggested, since different preventive interventions necessitate different interpretations even with similar development of PPH. [10, 18]

We calculated age-standardized PPH rates with direct method of standardization. We assessed stability of PPH, i.e. how annual PPH rates predicted the rates during the next year, with time series analysis. To assess geographic variation of these rates between the small areas, group-based trajectory models [19] were applied: 1) a basic model covered the variation of PPH as a single indicator, and 2) the multitrajectory model focused on the variation of the three subtypes of PPH (acute, chronic, and vaccine-preventable causes). Thus we calculated annual PPHs for each small area, and grouped them by the slopes and levels of their PPH. Before the analysis, based solely on the deductions of the researchers, we estimated that the number of groups in analysis would be between 6-9. To adjust this approximation, we applied BIC (Bayesian information criteria), LMR-LRT (Lo-Mendell-Rubvin likelihood ratio test) and entropy.

To further understand the differences between the groups of small areas, we added several types of area-level explanatory factors in the analysis: 1) healthcare-dependant factors, 2) populations sociodemographic characteristics, 3) municipal characteristics, and 4) other explanatory factors.

1) waiting times to GPs consultation, vacant GP posts, GP workload (Falster et. al. has used GP consultations divided by occupied GP posts [10])

2) gender, proportion of unemployed, proportion of those living alone, medicines reimbursed by National Health Insurance, polarization of the areas inhabitants by income/education/ethnicity (Index of Concentration - ICE)

3) municipal structure/type (individual municipality, consolidation of municipalities or host municipality), degree of urbanization, hospital

district, proportion of the lowest income quintile (as an indicator for disadvantaged municipalities)

Outcomes

- definition for small areas will be health centre districts, for which we shall calculate age-standardized PPH rates by genders and three PPH subgroups: acute, chronic, and vaccine-preventable

Figures and tables:

1. Table: direct frequencies or cross tabulation of PPH-rates in three (or four) years: 1996, 2004 and 2013
   - total rates and rates for each subgroup (acute, chronic, and vaccine-preventable)
   - gender
   - age-groups (40-64, 65-74, and +75)
   - distribution by the degree of urbanization of health centre districts
   - map to illustrate over time development of PPH rates

2. Figure: a line plot to begin with, from which we shall further decide the design of the final plot [box-plot, dot-plot, or compilation of both?]
   - y-axis (PPH rate), x-axis (year), variable (health centre district)
     - median, quartiles, and extreme values (box-plot)
   - http://www.bloodjournal.org/content/122/5/629?sso-checked=true
   - another option would be a dot-plot + line-plot
     - median, quartiles, and maybe extreme values
   - from this figure reader would see the possible turning points in PPH development

3. Figure or table on the development of PPH in 20 hospital districts (multilevel model?)
   - both the total PPH rate and the three subgroups (mentioned above)
   - four levels: year, individual (artificial level), health centre district, and hospital district
   - with this we try to clarify "what is the driving factor for PPH rates, and from which level it arises from?" – in other words do the different operation practises of the hospitals cause systematic geographical differences or do they mainly occur in the level of either individuals or health centre districts?

4. Table on the geographical stability of PPH – how do the PPH rates of previous year forecast the rates of the next year(s)?
    - multilevel model as a lagged-analysis, from which we try to estimate autocorrelations
    - time series analysis/ autoregressive analysis, or autoregressive coefficient?
    - does this require grouping of health centre districts into hospital districts, or could they be presented independently?

5. Figure considering the trajectory groups of health centre districts – and the development of their PPH
    - health centre districts will be distributed to trajectories with group-based trajectory model (GBTM) by the level and development of their PPH rates
    - basic model: total PPH rates
    - multitrajectory model: PPH rates by three subgroups (acute, chronic, vaccine-preventable)
    - assessment of effect of explanatory variables: why certain health care districts are allocated on specific trajectories?
    - explanatory factors are area-level variables
    - population structure as a categorising factor (needs to be aggregated from individual data)

6. Table comprising the list of PPH diagnoses, included as an Appendix

If this analysis works and significant geographical differences in levels or trends of PPH rates emerges, we could send these results to municipal administrations or primary care officials of the municipalities. (Unless the results are really obvious/ already regarded as "public knowledge").

References

1. Billings J, Anderson GM, Newman LS: **Recent findings on preventable hospitalizations.** Health Aff (Millwood) 1996, **15**(3):239-249.

2. Ansari Z: **The concept and usefulness of ambulatory care sensitive conditions as indicators of quality and access to primary health care.** Australian Journal of Primary Health 2007, **13**(3):91-110.

3. Eggli Y, Desquins B, Seker E, Halfon P: **Comparing potentially avoidable hospitalization rates related to ambulatory care sensitive conditions in Switzerland: the need to refine the definition of health conditions and to adjust for population health status.** BMC Health Serv Res 2014, **14**:25-6963-14-25.

4. Rosano A, Loha CA, Falvo R, van der Zee J, Ricciardi W, Guasticchi G, de Belvis AG: **The relationship between avoidable hospitalization and accessibility to primary care: a systematic review.** Eur J Public Health 2013, **23**(3):356-360.

5. Gibson OR, Segal L, McDermott RA: **A systematic review of evidence on the association between hospitalisation for chronic disease related ambulatory care sensitive conditions and primary health care resourcing.** BMC Health Serv Res 2013, **13**:336-6963-13-336.

6. Magan P, Otero A, Alberquilla A, Ribera JM: **Geographic variations in avoidable hospitalizations in the elderly, in a health system with universal coverage.** BMC Health Serv Res 2008, **8**:42.

7. Borda-Olivas A, Fernandez-Navarro P, Otero-Garcia L, Sanz-Barbero B: **Rurality and avoidable hospitalization in a Spanish region with high population dispersion.** Eur J Public Health 2013, **23**(6):946-951.

8. Berlin C, Busato A, Rosemann T, Djalali S, Maessen M: **Avoidable hospitalizations in Switzerland: a small area analysis on regional variation, density of physicians, hospital supply and rurality.** BMC Health Serv Res 2014, **14**:289-6963-14-289.

9. Burgdorf F, Sundmacher L: **Potentially avoidable hospital admissions in Germany: an analysis of factors influencing rates of ambulatory care sensitive hospitalizations.** Dtsch Arztebl Int 2014, **111**(13):215-223.

10. Falster MO, Jorm LR, Douglas KA, Blyth FM, Elliott RF, Leyland AH: **Sociodemographic and health characteristics, rather than primary care supply, are major drivers of geographic variation in preventable hospitalizations in Australia.** Med Care 2015, **53**(5):436-445.

11. Magan P, Alberquilla A, Otero A, Ribera JM: **Hospitalizations for ambulatory care sensitive conditions and quality of primary care: their relation with socioeconomic and health care variables in the Madrid regional health service (Spain).** Med Care 2011, **49**(1):17-23.

12. Orueta JF, Garcia-Alvarez A, Grandes G, Nuno-Solinis R: **Variability in potentially preventable hospitalisations: an observational study of clinical practice patterns of general practitioners and care outcomes in the Basque Country (Spain).** BMJ Open 2015, **5**(5):e007360-2014-007360.

13. Erhola M, Jonsson PM, Pekurinen M, Teperi J: **Jonottamatta hoitoon.** THL: n aloite perusterveydenhuollon vahvistamiseksi. National Health Institute, 2013.

14. Mattila Y: *Suuria käännekohtia vai tasaista kehitystä? : tutkimus Suomen terveydenhuollon suuntaviivoista:* Helsinki: Kelan tutkimusosasto; 2011.

15. Parmanne P, Ruskoaho J, Vänskä J: **Physicians in Finland, Statistics on physicians and the health care system 2014.** 2015.

16. Purdy S, Griffin T, Salisbury C, Sharp D: **Ambulatory care sensitive conditions: terminology and disease coding need to be more specific to aid policy makers and clinicians.** Public Health 2009, **123**(2):169-173.

17. Page A, University of Adelaide. Public Health Information Development Unit., Australian Institute of Health and Welfare.: *Atlas of avoidable hospitalisations in Australia : ambulatory care-sensitive conditions:* Adelaide, S. Aust.: Public Health Information Development Unit, the University of Adelaide; 2007.

18. Saver BG, Wang CY, Dobie SA, Green PK, Baldwin LM: **The central role of comorbidity in predicting ambulatory care sensitive hospitalizations.** Eur J Public Health 2014, **24**(1):66-72.

19. Nagin DS, Odgers CL: **Group-based trajectory modeling in clinical research.** Annu Rev Clin Psychol 2010, **6**:109-138.
